# The dilemma of the gender assignment in a Portuguese adolescent with disorder of sex development due to 17β-hydroxysteroid-dehydrogenase type 3 enzyme deficiency

**DOI:** 10.1530/EDM-14-0064

**Published:** 2014-09-01

**Authors:** Carla Costa, Cíntia Castro-Correia, Alda Mira-Coelho, Bessa Monteiro, Joaquim Monteiro, Ieuan Hughes, Manuel Fontoura

**Affiliations:** Paediatric Endocrinology and Diabetology Unit Department of Paediatrics, Faculty of Medicine of Porto, Hospital São João, Hernãni Monteiro, Porto, 4202-451, Portugal; 1 Department of Psychiatry, Hospital São João, Porto, Portugal; 2 Department of Paediatric Surgery, Hospital São João, Porto, Portugal; 3 Endocrinology and Diabetology Unit, Department of Paediatrics, Addenbrook's Hospital, University of Cambridge, Cambridge, UK

## Abstract

The development of male internal and external genitalia in an XY fetus requires a complex interplay of many critical genes, enzymes, and cofactors. The enzyme 17β-hydroxysteroid-dehydrogenase type 3 (17βHSD3) is present almost exclusively in the testicles and converts Delta 4-androstenodione (Δ4) to testosterone. A deficiency in this enzyme is rare and is a frequently misdiagnosed autosomal recessive cause of 46,XY, disorder of sex development. The case report is of a 15-year-old adolescent, who was raised according to female gender. At puberty, the adolescent had a severe virilization and primary amenorrhea. The physical examination showed a male phenotype with micropenis and blind vagina. The Tanner stage was A3B1P4, nonpalpable gonads. The karyotype revealed 46,XY. The endocrinology study revealed: testosterone=2.38 ng/ml, Δ4>10.00 ng/ml, and low testosterone/Δ4 ratio=0.23. Magnetic resonance imaging of the abdominal–pelvic showed the presence of testicles in inguinal canal, seminal vesicle, prostate, micropenis, and absence of uterus and vagina. The genetic study confirmed the mutation p.Glu215Asp on *HSD17B3* gene in homozygosity. The dilemma of sex reassignment was seriously considered when the diagnosis was made. During all procedures the patient was accompanied by a child psychiatrist/psychologist. The teenager desired to continue being a female, so gonadectomy was performed. Estrogen therapy and surgical procedure to change external genitalia was carried out. In this case, there was a severe virilization at puberty. It is speculated to be due to a partial activity of 17βHSD3 in the testicles and/or extratesticular ability to convert Δ4 to testosterone by 17βHSD5. Prenatal exposure of the brain to androgens has increasingly been put forward as a critical factor in gender identity development, but in this case the social factor was more important for the gender assignment.

**Learning points:**

- In this case, we highlight the late diagnosis, probably because the patient belongs to a poor family without proper primary medical care.
- We emphasize the psychological and social aspects in the sex assignment decision.

## Background

Disorder of sex development (DSD), is defined as a congenital condition in which development or chromosomal, gonadal, or anatomical sex is atypical, therefore 46,XY DSD refers to a condition in which a child has a 46,XY karyotype, but in whom gonadal or anatomical sex is atypical (1) (2) (3).

The development of the male internal and external genitalia in an XY fetus requires a complex interplay of many critical genes, enzymes, and cofactors (4). The lack of one of these critical factors can lead to a child with a 46,XY DSD (1).

The enzyme 17β-hydroxysteroid dehydrogenase type 3 (17βHSD3) is present almost exclusively in the testes and converts Delta 4-androstenodiona (Δ4) to testosterone. A deficiency in the 17βHSD3 enzyme is rare, and frequently misdiagnosed as autosomal recessive cause of 46,XY DSD. This disorder was first described in 1971 (5). 17βHSD3 enzyme deficiency, previously termed 17-ketosteroid reductase deficiency, is the most common testosterone biosynthesis defect of 46,XY DSD (1). Deficiency in the 17βHSD3 enzyme can be caused by either homozygous or compound heterozygous mutations in the *HSD17B3* gene (6). Mutations in the *HSD17B3* gene confer a spectrum of 46,XY disorders of sexual organ development, ranging from completely undervirilized external female genitalia, predominantly female, ambiguous, to predominantly male with micropenis and hypospadias. The most frequent presentation of 17βHSD3 deficiency is a 46,XY individual with female external genitalia, labial fusion, and a blind ending vagina, with or without clitoromegaly.

The diagnosis can be easily missed in early childhood as the clinical presentation may be subtle. If the condition is not diagnosed early, patients present with severe virilization and primary amenorrhea in adolescence and may undergo a change from female to male gender role. A low testosterone/Δ4 ratio on baseline or human chorionic gonadotropin (hCG) stimulation testing is suggestive of 17βHSD3 deficiency. The diagnosis can be confirmed by molecular genetic studies.

## Case presentation

**Figure 1:**
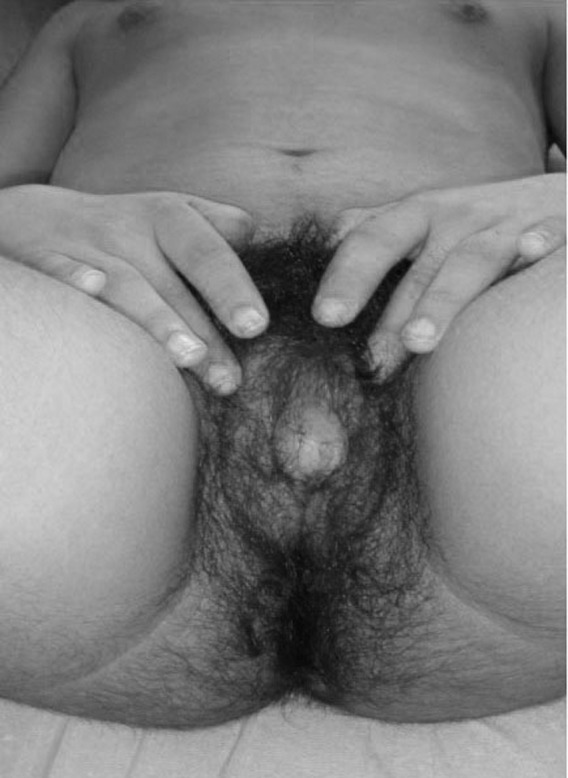
Physical examination of the 17βHSD3 deficiency patient.

A 15-year-old adolescent was raised according to female gender. The patient had no significant past medical or surgical history. The family history was negative, non-consanguineous parents. At puberty the adolescent had primary amenorrhea and a severe virilization with deep voice, masculine muscular development, increased growth of body, and facial hair. The physical examination showed a male phenotype with micropenis and blind vagina. Her breasts were Tanner stage 1, pubic and axilar hair was Tanner stage 4 with nonpalpable gonads (Fig. 1). The karyotype revealed 46,XY. An endocrinology study (Table 1) revealed testosterone level: 2.38 ng/ml; Δ4>: 10.00 ng/ml; low testosterone/Δ4 ratio:0.23; dihydrotestosterone:0.127 ng/ml; testosterone/dihydrotestosterone ratio:18.7, and the urinary steroids: normal. Magnetic resonance imaging of abdominal–pelvic showed the presence of testicles in inguinal canal, seminal vesicle, prostate, and micropenis and the absence of uterus and vagina. Treatment with gonadotrophin-releasing hormone (GnRH) analog and flutamide was started in the first year, with good results. The genetic study confirmed the mutation p.Glu215Asp on gene *HSD17B3* in homozygosity.

**Table 1:** Chronological evolution of serial steroid hormone and gonadotrophin determinations

	**Peripheral venous blood concentrations**		
Pre-treatment	6 months post-treatment^*^	Normal range
Testosterone (ng/ml)	2.38	0.11	0.06–0.82
Δ4 (ng/ml)	>10.00	0.44	0.3–3.3
Testosterone/Δ4 ratio	<0.23		>0.8
DHEA-S (μg/dl)	227.1	157.1	65.1–368
17-OH-P (ng/ml)	1.07	0.87	0.60–2.60
DHT (ng/ml)	0.127		0.08–0.33
Testosterone/DHT ratio	18.7		<20
E_2_ (pg/ml)	20.7	13.2	8–40
LH (mUI/ml)	19.7	1.48	1.5–9.0
FSH (mUI/ml)	8.26	2.81	2.0–9.2
AST/ALT (UI/l)	24/21	27/20	10–31

Δ4, androstenodione; 17-OH-P, 17-hydroxy-progesterone; DHT, dihydrotestosterone; E_2_, estradiol; LH, luteinizing hormone; FSH, follicle-stimulating hormone.

* treatment with flutamide and GNRH analog.

The decisions about the management of the present case were made after interviews with the adolescent and her parents and in consultations among the endocrinologist, the surgeon, and the psychiatrist. During all procedures, the patient was accompanied by a child psychiatrist/psychologist.

**Figure 2:**
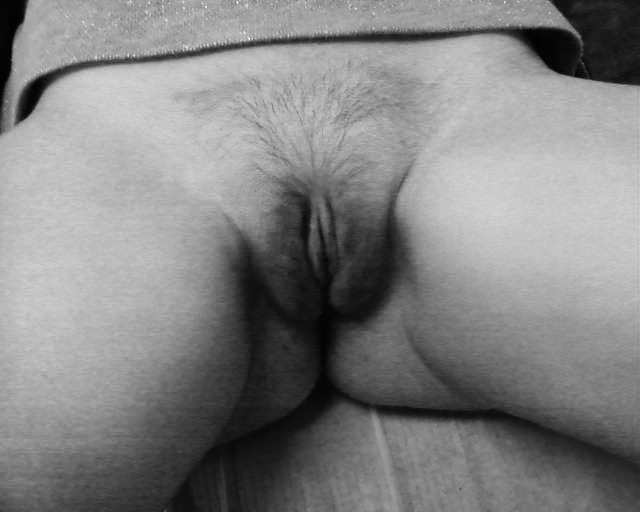
Patient after surgery.

The clinical course, the hormonal profile, and the karyotype (46,XY) were compatible with the diagnosis of 17β HSD3 deficiency, which was confirmed by DNA analysis. Upon discussing the possibilities for gender assignment, the father and the adolescent strongly opposed gender reassignment, expressing considerable fear of the social repercussions, especially considering the fact that they were members of a very small community. The teenager showed the desire to continue being a female, because she identified herself as a female. The pediatric surgeon explained all the procedures and the fact that if she wants to continue as a female it would eliminate the possibility of fertility. At the age of 16 years, gonadectomy was performed along with the surgical procedures to change external genitalia (Fig. 2). At this time, the patient stopped treatment with GNRH analog and flutamide and started to undergo estrogen replacement therapy. The microscopical examination of the testes showed normal testicular histology. No genetic analysis was made in the removed gonads.

After surgery and estrogen therapy she returned for a follow-up visit in a pleasant mood, more talkative, and dressed in very feminine clothes. She had breasts development under estrogen therapy. She did not express any regret about the surgical intervention, reported that her mood is consistently good and that socialization had significantly improved. She was no longer stigmatized, which is traumatic for DSD people.

## Discussion

In the literature, 28 mutations in the *HSD17B3* gene have been reported to date. The mutation p.Glu215Asp was found in three children (white Brazilian and English) with 46,XY DSD, one female with ambiguous genitalia at birth, male behaviors in child, and pubertal virilization, absence of menses and male gender role in another (7).

The risk of misdiagnosis is especially problematic because the clinical findings in 17βHSD3 deficiency may mimic androgen insensivity syndrome in childhood and 5α-reductase deficiency in puberty. Thus, correct diagnosis should be made early so that treatment, management, and genetic counseling can be specifically directed toward 17βHSD3 deficiency.

The patients with mutation in the *HSD17B3* gene may go unnoticed at birth since female external genitalia is common (8). These children are usually assigned the female gender and raised as such. In this patient, the diagnosis was missed until adolescence. Those who come to medical attention in childhood have some degree of virilization or inguinal hernia with testes present along the inguinal canals or labioscrotal folds. Less frequently, micropenis or hypospadia have also been reported. In this patient, the female gender was assigned at birth and she was raised accordingly. It is interesting to know that she probably had medical attention in childhood but no one detected any degree of virilization.

At the time of puberty, patients initially raised as females and who have not undergone gonadectomy display primary amenorrhea, varying degrees of virilization including development of male body habitus, increased body hair, and deepening of the voice; in some individuals, it prompts a change to a male gender role. This has been speculated to be due to the partial activity of 17βHSD3 in the testes and extratesticular testosterone conversion by other members of the family, such as 17βHSD5 (8).

Certain XY individuals with deficiency of the enzyme 17βHSD3 and raised as females ask for male reassignment after puberty (9). Why some of these individuals change their gender role late in life and others do not is an unresolved question which might be related to prenatal androgen effects on the developing brain or to social and emotional parameters. It is very important to establish a specific diagnosis early on. This is underlined by the present case, which highlights the need for early intervention so that virilization in those raised as females can be prevented. The virilization is difficult to completely reverse, especially regarding changes in voice and body build.

## Conclusion

The 17βHSD3 deficiency causes an autosomal recessive form of 46,XY DSD that can be clinically indistinguishable from other forms. The correct diagnosis can be achieved by systematic endocrine evaluation and, most importantly, by the calculation of the testosterone/Δ4 ratio. Molecular genetic testing confirms the diagnosis and provides the orientation for genetic counseling. Prenatal exposure of the brain to androgens has increasingly been put forward as a critical factor in gender identity development, but in this case the social factor was more important for the gender assignment.

## Funding statement

This research did not receive any specific grant from any funding agency in the public, commercial or not-for-profit sector.

## Patient consent

Written informed consent has been obtained from the patient for publication of the submitted article and accompanying images.

## Author contribution statement

Prof. I Hughes supervised the reported case from the beginning, including the genetic study and clinical tests. The Genetic Department of Hospital São João handled the connection with the Molecular Laboratory of University College London Hospital. The Pathology Department of Hospital São João performed the histological exam of the testes. And finally, the Molecular Laboratory of University College London Hospital performed the genetic study.
